# Construction of Photoelectrochemical DNA Biosensors Based on TiO_2_@Carbon Dots@Black Phosphorous Quantum Dots

**DOI:** 10.3390/mi12121523

**Published:** 2021-12-08

**Authors:** Kai Song, Jianwei Lin, Yafeng Zhuang, Zhizhong Han, Jinghua Chen

**Affiliations:** 1 School of Pharmacy, Fujian Medical University, Fuzhou 350122, China; songkai1396@126.com (K.S.); a15260712344@163.com (J.L.); 18760367357@139.com (Y.Z.); cjh_huaxue@fjmu.edu.cn (J.C.); 2 Fujian Key Laboratory of Drug Target Discovery and Structural and Functional Research, Fuzhou 350122, China

**Keywords:** titanium dioxide, carbon dots, black phosphorus quantum dots, photoelectrochemical biosensor

## Abstract

In this work, carbon dots (CDs) and black phosphorus quantum dots (BPQDs) were used to decorate titanium dioxide to enhance the photoelectrochemical (PEC) properties of the nanocomposites (TiO_2_@CDs@BPQDs), and the modified nanocomposites were used to sensitively detect DNA. We used the hydrothermal method and citric acid as a raw material to prepare CDs with good dispersion and strong fluorescence properties. BPQDs with a uniform particle size were prepared from black phosphorus crystals. The nanocomposites were characterized by fluorescence spectroscopy, UV-Vis absorption spectroscopy, Fourier transform infrared spectroscopy (FTIR) and transmission electron microscopy (TEM). The preparation method of the working electrode was explored, the detection conditions were optimized, and the sensitive detection of target DNA was achieved. The results demonstrate that CDs and BPQDs with good optical properties were successfully prepared, and they were successfully combined with TiO_2_ to improve the PEC performance of TiO_2_@CDs@BPQDs. The TiO_2_-based PEC DNA detection method was constructed with a detection limit of 8.39 nM. The constructed detection method has many advantages, including good sensitivity, a wide detection range, and good specificity. This work provides a promising PEC strategy for the detection of other biomolecules.

## 1. Introduction

Photoelectrochemical (PEC) detection technology has been used in biomolecule detection for a long time and has good application prospects in environmental monitoring and biomedical diagnosis. The light response materials in PEC sensors are the core part of the whole technology, usually adopting semiconductor materials. TiO_2_ is a type of low toxicity, environmental protection material, has good photostability and photosensitivity, and has been widely used in many fields, such as solar cells [1], photocatalytic degradation of pollutants [2], and PEC sensors [3].

However, as TiO_2_ belongs to n-type wide bandgap semiconductors, the photogenerated electrons and holes are easy to recombine. In addition, it only absorbs ultraviolet light in sunlight, which only accounts for 5% of the energy in sunlight [4,5]. These properties indicate that the photochemical properties of TiO_2_ are not good, which limits its application in PEC detection to a large extent. TiO_2_ can be modified by the following methods to improve its PEC properties, including non-metal or metal ion doping [6,7], semiconductor composite [8], surface sensitization [9], and precious metal deposition [10]. In order to ensure the low toxicity of the material after modification, two kinds of narrow-gap semiconductors, carbon dots (CDs) and black phosphorous quantum dots (BPQDs), were used to decorate TiO_2_ in this work.

CDs are a kind of carbon-based zero-dimensional nanometer material that is composed of a carbonaceous framework and surface groups with a particle size of less than 10 nm. It has good fluorescence performance, low toxicity, good biocompatibility, a tunable emission wavelength, easy functionalization and up-conversion photoluminescence [11], etc. In combination with carbon dots, the light absorption range of TiO_2_ can be extended from the ultraviolet light region to the visible light region to, thereby, achieve the purpose of improving its PEC properties. 

Black phosphorus (BP) is a special 2D material with tunable bandgap and has been applied in the field of photonics and electronics [12]. However, BPQDs are quasi-zero-dimensional nanomaterials, and the sizes are all nanoscale in three dimensions. The energy level structure of BPQDs is discrete due to the quantum confining effect, which is similar to the properties of the atomic spectrum. 

BPQDs have many excellent performance traits, such as good fluorescence properties, a controllable energy bandgap [13] and a relatively high carrier mobility rate [14], and they can inhibit carriers of radiative transition. At the same time, due to the quantum confinement effect and the existence of the edge effect [15], the PEC performance is superior to other related materials of black phosphorus, for example, phosphorene and so on. Therefore, it has a very good application prospects in the modification of the PEC properties of TiO_2_.

In this work, CDs and BPQDs were used to decorate TiO_2_ and improve the PEC performance of the nanocomposites (TiO_2_@CDs@BPQDs), and the PEC biosensors based on TiO_2_@CDs@BPQDs were constructed for the detection of DNA with high detection sensitivity while maintaining the low toxicity and environmental protection in the process of detection.

## 2. Experimental

### 2.1. Materials and Reagents

Citric acid (CA, ≥ 99.5%), polyethylene glycol diamine (H_2_N-PEG-NH_2_, 97%), polyethylene polyamine (AR), and titanium dioxide (P25) were purchased from Shanghai Macklin Biochemical Co., Ltd. (Shanghai, China). Natrium hydroxydatum (NaOH, AR) was obtained from Shanghai Hushi Laboratory Equipment Co., Ltd. (Shanghai, China). Black phosphorus powder (≥99.99%) was bought from Jiangsu Xianfeng Nanomaterial Technology Co., Ltd. (Jiangsu, China). Cellulose ester dialysis membranes (500–1000Da) were purchased from Shanghai Yuanye Biotechnology Co., Ltd. (Shanghai, China). ITO substrates were obtained from Foshan Meijingyuan Glass Technology Co., Ltd. (Guangdong, China). 

Absolute ethyl alcohol (C_2_H_5_OH, AR) and acetone (CH_3_COCH_3_, AR) were purchased from Sinopharm Chemical Reagent Co. Ltd. (Shanghai, China). Phosphate buffer saline (PBS (1X), 0.0067M), N-methyl pyrrolidone (NMP, ≥99.0%), bovine serum albumin (BSA, 20mg/mL), and DNA were acquired from Sangon Bioengineering Co., Ltd. (Shanghai, China). The sequence of the used DNA was as follows: 5′-AAAAAAAAGATTGCATG-3′ (probe DNA), 5′-CTAACGGTACAAAAAA-3′ (target DNA). All reagents were of analytical grade and were used without further purification.

### 2.2. Apparatus

The UV–Vis absorption spectrum was obtained with a Genesys 150 UV-Visible spectrophotometer (ThermoFisher Scientific Inc., Waltham, MA, USA). The fluorescence spectrum was detected by F96PRO fluorescence spectrometer (Lengguang, Shanghai, China) with an excitation wavelength of 350 nm. HSX-F/UV 300 xenon lamp (NBeT, Beijing, China) was used as excitation source for the PEC characterization. The electrochemical experiments were carried on at room temperature utilizing an electrochemical workstation (CHI660D, CH Instruments, Shanghai, China) with a three-electrode mode: ITO loaded with the as-prepared nanocomposites was used as the working electrode, with Ag/AgCl as the reference electrode, and platinum wire as the counter electrode. 

The morphology and particle size of CDs and BPQDs were observed using Tecnai G2 transmission electron microscopy (TEM. FEI, Hillsboro, OR, USA) with an acceleration voltage of 200 kV. The surface molecular structures of CDs and BPQDs were characterized by a Fourier transform infrared spectrometer (Perkin Elmer Spectrum 2000, Perkin Elmer, Waltham, MA, USA).

### 2.3. General Synthetic Method for Carbon Dots (CDs)

The specific synthesis process for CDs was as follows. We dissolved 0.84 g citric acid and 0.2 g polyethylene diamine in 20 mL ultra-pure water, and then 536 μL of polyethylene polyamine was added to the solution and mixed evenly. After reacting for 5 h at 200 °C, it was naturally cooled to room temperature. The resulting brown liquid was filtered with a 0.2 μm microporous filtration film, moved to a dialysis bag with 500–1000 trapped molecular weight, and the water was replaced every 6–8 h for 72 h to remove excess raw materials and coexisting small molecules from the reaction. The carbon point solution concentration was 10.45 mg·mL^−1^.

### 2.4. Synthesis of Black Phosphrous Quantum Dots (BPQDs)

BPQDS was synthesized in the method developed by previous works [16]. In brief, 10 mg black phosphate crystal powder and 1 mL NMP were added into a 100 mL beaker and fully ground with a glass bar until the solution became black. Then, 100 mg NaOH and 99 mL NMP were added to the beaker and mixed. The mixture was transferred to 150 mL three-neck flask, reacted for 6 h under nitrogen protection at 140 °C. Finally, the resulting solution was centrifuged at 7000 rpm for 20 min at room temperature. The supernatant was collected and centrifuged again (12,000 rpm) for 20 min at room temperature. The precipitate was collected and a few solvents were retained; therefore, a BPQD NMP suspension was obtained and kept at 4 °C.

BPQDs should be redispersed into the water before use. A 0.1 mL BPQDs NMP suspension was mixed with 0.4 mL ultrapure water, and the mixture was centrifuged at 12,000 rpm for 10 min at room temperature. Then, the supernatant was discarded, and we added 0.2 mL ultrapure water to prepare a BPQDs aqueous solution.

### 2.5. Synthesis of TiO_2_@CDs Composites

Commercial P25 was used as the TiO_2_ source. First, 10 mL of a 3 mg/mL TiO_2_ solution and 10 mL of a CDs solution were added to a 50 mL single-neck flask, stirred at 60 °C for 2 h. The resulting solution was centrifuged at 7000 rpm for 10 min, the upper clear liquid was then discarded, and 20 mL superpure water was redispersed to precipitate to obtain the TiO_2_@CDs composite product.

### 2.6. Fabrication of TiO_2_@CDs@BPQDs Electrode

ITO substrates were cleaned stepwise with sonication in detergent, acetone, ethanol, and deionized water, and dried in an oven. The cleaned ITO electrodes were stored in anhydrous ethanol. The composite electrode was prepared as follows. First, 50 μL TiO_2_@CDs solution was dripped with a pipette onto the dry ITO surface, expanded naturally, and dried at room temperature. Secondly, 10 μL BPQDs aqueous solution was slowly dripped onto the electrode surface and naturally dried at room temperature to obtain the TiO_2_@CDs@BPQDs (TCPs) electrode. Note that the drip coating area of the surface of each electrode should be as consistent as possible.

### 2.7. Detection of the Target DNA

The probe DNA (P-DNA) used in this experiment was modified with -NH_2_ at the 5′ terminus. Then, the P-DNA can be connected to TCPs nanocomposites through the amino group and the carboxyl group on the surface of CDs or the phosphate group of BPQDs. First, 10 μL 100 nM P-DNA dripped to the composite electrode and slowly spread, incubated at 37 °C for 1 h, and then washed three times with Tris-HCl solution. 

Thus, the P-DNA/TiO_2_@CDs@BPQDs nanocomposites (marked as P/TCPs) electrodes were prepared. Following, 20 μL 0.5% BSA solution dripped to the electrode surface and incubated for 2 h at room temperature, which was called BP/TCPs. Finally, 10 μL of target DNA (T-DNA) solution was added onto the electrode and incubated at 37 °C for 1 h, and then the electrode was cleaned three times with Tris-HCl solution. The ultimate electrodes were named TBP/TCPs electrodes. The concentration of T-DNA was varied from 10 to 130 nM with an interval of 10 nM.

As Figure S1 shows, the PEC detection was carried out under simulated sunlight irradiation with a xenon lamp. The PEC signal was recorded using a traditional three-electrode system with an electrochemical workstation. In this detection system, the prepared electrode acted as working electrode, the saturated Ag/AgCl electrode worked as reference electrode, and the platinum wire electrode was used as auxiliary electrode. The working potential was 0 V. Additionally, the working area was about 1 × 0.7 cm^2^. The electrolyte was PBS solution (pH = 7.06). The cyclic voltammetry was also measured with the three-electrode system. The potential range was set from −0.30 to 0.80 V, and the scanning rate was 0.05 V/s.

## 3. Results and Discussion

### 3.1. Construction of Biosensors Based on TiO_2_@CDs@BPQDs Nanocomposites

**Scheme 1:**
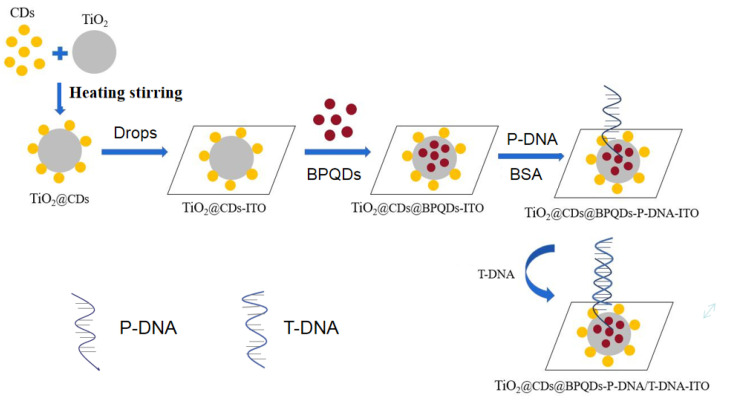
Schematic constructed process of the biosensors based on TCPs nanocomposites.

As Scheme 1 shows, TCPs were used to modify ITO electrodes by drip coating, using the good optical properties of CDs and BPQDs to enhance the PEC properties of the composite. The composite surface is rich in carboxylic, phosphate groups and other groups, which can effectively combine with the amino groups of P-DNA. Utilizing complementary base pairing, T-DNA and P-DNA combine to form a double helix structure. Both nucleic acid and protein have relatively large resistance, adsorb to the surface, and will inhibit the transfer of photogenerated electrons leading to a decrease of photocurrent. Therefore, quantitative analysis of T-DNA can be realized through a photocurrent.

### 3.2. Characterization of the CDs

**Figure 1:**
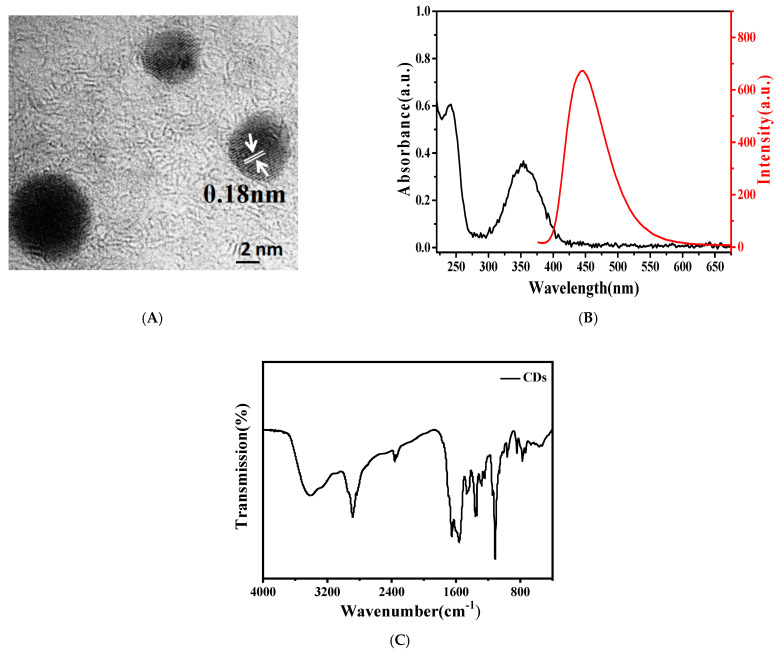
(**A**) Transmission electron microscopy (TEM) image, (**B**) UV-Vis absorption spectrum and fluorescence spectrum, and (**C**) Fourier transform infrared spectrum (FTIR) of carbon dots (CDs).

Morphological analysis of CDs is shown in Figure 1A. The TEM characterization results exhibit that the size of the prepared CDs is about 5 nm with a lattice stripe spacing of 0.18 nm, corresponding to the (102) crystal plane of the graphite carbon [17]. The spectral characterization results are shown in Figure 1B. The fluorescence property used 350 nm as the excitation wavelength. CDs have a maximum emission peak at 438 nm and have strong fluorescence intensity. The UV-Vis light absorption spectrum of CDs demonstrated that the prepared CDs had two distinct absorption peaks at 240 and 354 nm, and the absorption peak at 240 nm belongs to the π−π * transition of C=C, while the absorption peak at 354 nm likely belongs to the n−π * electron transition of C=O [18]. 

The molecular structure of CDs can be further determined by Fourier transform infrared spectrum (FTIR). As shown in Figure 1C, the stretching vibration of O-H results in two characteristic peaks at 3400 cm^−1^ and 1013 cm^−1^, of which the stronger characteristic peak at 1013 cm^−1^ is due to much adsorbed water molecules at the surface. The stretching vibration of -CH group leads to a characteristic peak at 2950 cm^−1^. Furthermore, characteristic peaks occurring at 1660 cm^−1^ and 1368 cm^−1^ correspond to asymmetrical and symmetrical stretching vibrations of -COOH, respectively. 

While the peak of 1550 cm^−1^ belongs to the stretching vibration of C=C, and the characteristic peak at 1441 cm^−1^ belongs to the bending vibration of the N-H. The characteristic peak at 1154, 1295, and 1149 cm^−1^ belong to the stretching vibrations of the C-O, C-N, and C-C, respectively [19]. The results of infrared spectra show that the prepared CD surface is rich in various groups, where the presence of -COOH and N-H lays the foundation for their subsequent connection to other materials.

### 3.3. Characterization of BPQDs

**Figure 2:**
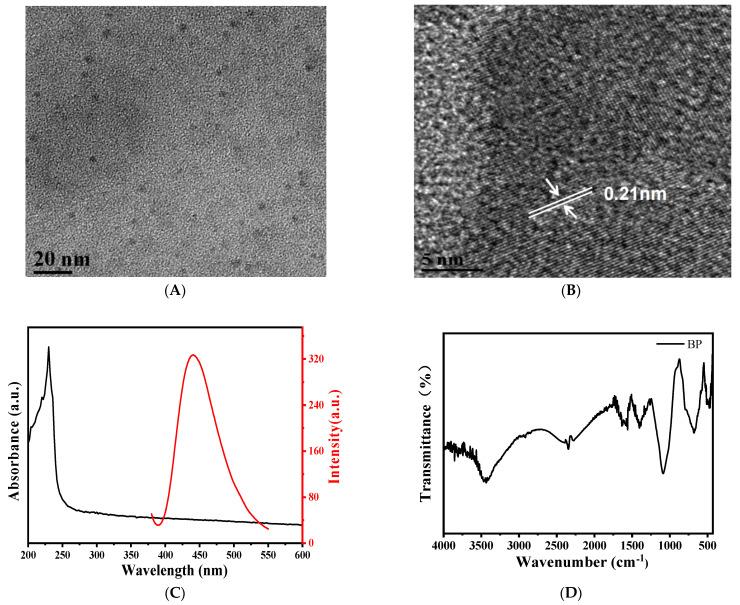
(**A**,**B**) TEM image, (**C**) UV-Vis absorption spectrum and fluorescence spectrum, (**D**) FTIR of BPQDs.

BPQDs were prepared with flux method from the black phosphorus crystal as the raw material. As NMP is a good organic solvent for stripping 2D materials [20] and the saturated NaOH NMP solution has been proven to improve the stability of BP nanosheets [21], the saturated NaOH NMP solution was chosen to prepare BPQDs. The prepared NMP solution of BPQDs was a black liquid with good dispersion as shown in Figure 2A,B. It can be seen that the prepared BPQDs had good dispersion and uniform particle size with an average particle size of about 2 nm. 

The results of HRTEM show that the lattice spacing was 0.21 nm, corresponding to the (040) crystal face of BP crystal [22]. As shown in Figure 2C, the maximum emission peak is found at 440 nm under the excitation wavelength of 350 nm, and the fluorescence intensity is satisfactory. The UV-Vis spectrum displays that the prepared BPQDs had good absorption in the visible region, which is beneficial to enhance the light absorption of the nanocomposites. We found that the characteristic FTIR peaks of phosphorus oxyacids or PO_x_ appeared between 600 cm^−1^ and 1750 cm^−1^ (Figure 2D), indicating that part of the BPQDs was oxidized.

### 3.4. Preparation and Characterization of Photoanode

**Figure 3:**
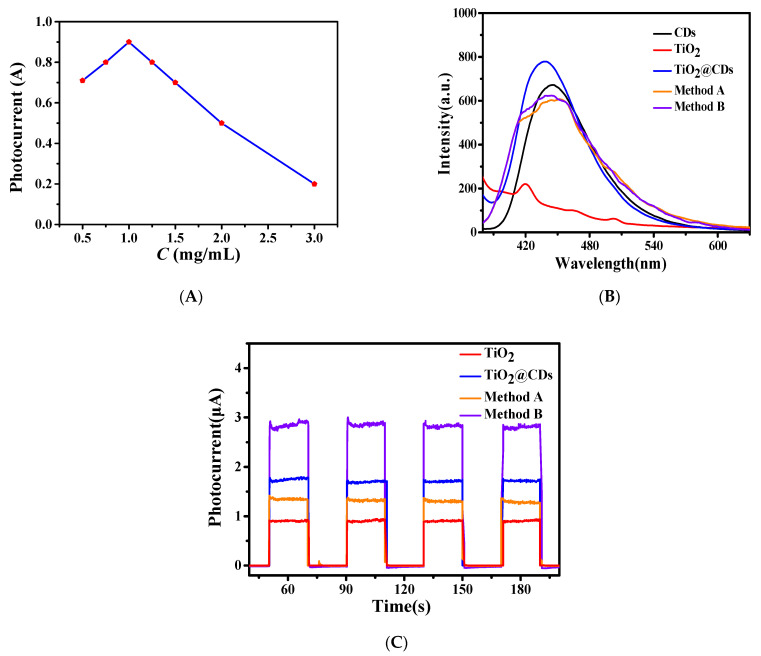
(**A**) Photocurrent of the electrodes prepared with different concentrations of TiO_2_, (**B**) fluorescence emission spectra, and (**C**) Photoelectrochemical (PEC) properties for different samples.

In this work, TiO_2_ nanoparticles (P25) were used as substrates with different concentrations. As shown in Figure 3A, we found that the TiO_2_ solution with the concentration of 1 mg/mL had the best PEC performance with the photocurrent value of 0.9A, and the photocurrent value decreased when the concentration was higher or lower. The reason may be that TiO_2_ itself can produce a photocurrent well. When the concentration increases, more TiO_2_ particles will participate in the photoreaction and produce more photocurrent. However, if the concentration is too high, it is easy to cause an agglomeration of particles, the interface resistance increases, and the photocurrent decreases. Therefore, the concentration of TiO_2_ in subsequent experiments is 1 mg/mL.

Two methods were used to prepare TiO_2_@CDs@BPQDs nanocomposites. Method A was to mix TiO_2_, CDs, and BPQDs at the same time, and stir for a certain time. Method B is that TiO_2_ and CDs were combined at first, and then added BPQDs into the mixture. The products synthesized were centrifuged at 12,000 rpm, and the precipitate was collected. As shown in Figure 3B, the fluorescence intensity of pure TiO_2_ is extremely weak. However, the fluorescence spectrum of TiO_2_@CDs is similar to that of pure CDs, but a certain blue shift occurs, indicating that TiO_2_ and CDs can directly form chemical bonds. 

The fluorescence intensity of the TiO_2_@CDs was enhanced compared with that of pure CDs, indicating that TiO_2_ and CDs have a good synergistic effect, which is conducive to the enhancement of photocurrent. In addition, whether BPQDs were combined with method A or B, the fluorescence emission spectrum decreased significantly compared with that of TiO_2_@CDs, the peak type changed slightly, and a shoulder peak appeared. As BPQDs and CDs both have good fluorescence performance, two fluorescence peaks could appear at the same time. The decrease of the intensity indicates that the BPQDs support enhances the electron-hole separation.

According to the analysis of Figure 3C, the PEC performance of TiO_2_ was significantly improved after CDs were loaded, from 0.9 A to 1.7 μA, which further demonstrates the successful combination of TiO_2_@CDs. Compared with TiO_2_@CDs, the photocurrent of the nanocomposites prepared by method A decreased to 1.3 μA, while the photocurrent of the product with method B increased to 2.7 μA. 

The possible reason for the two differences is that, in method A, the products obtained by stirring the three materials together may cause the BPQDs to have a high degree of recombination resulting in the aggregation of BPQDs in the composite electrode, which greatly promotes the probability of electron-hole recombination and, thus, weakens the photocurrent [23]. In method B, BPQDs were added at last, and BPQDs and CDs formed contacts on the nanoscale. Under the action of grating pressure effect, the built-in electric field promotes the hole injecting into TiO_2_, realizing the growth of photocurrent [24]. As Figure S2 shows, the size of the TiO_2_@CDs@BPQDs nanocomposites was larger than 200 nm with irregular shapes.

### 3.5. Construction and Condition Optimization of the Developed Biosensors

**Figure 4:**
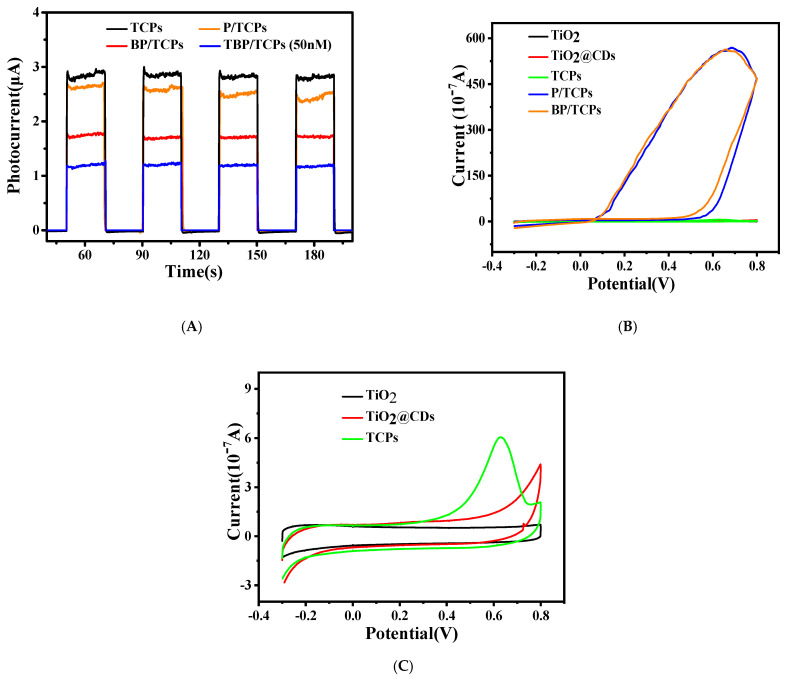
(**A**) PEC characterization, (**B**,**C**) CV curves of the different electrodes.

As shown in Figure 4A, the photocurrents of the prepared TCPs electrode after loading P-DNA and BSA solution were reduced significantly, from 2.8 to 2.6 and 1.75 μA, respectively, which is in line with expectations, since the P-DNA and BSA are insulators with poor conductivity. After the addition of 10 μL 50 nM T-DNA, the photocurrent decreased to 1.2 μA, indicating that the developed biosensor was effective to detect the T-DNA. Figure 4B is the CV curve of different samples, and Figure 4C is the local amplification of Figure 4B. It can be seen from the figures that neither TiO_2_ nor TiO_2_@CDs had a redox peak, while the TCPs had an obvious oxidation peak, which belongs to the oxidation peak of BPQDs. After the addition of P-DNA and BSA, the oxidation peak disappeared and a large circulation ring appeared, indicating that P-DNA and BSA successfully covered the electrode surface.

**Figure 5:**
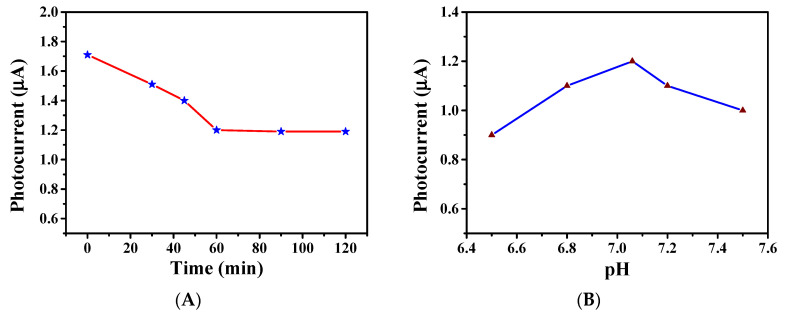
Factors affecting the PEC performance of the constructed electrode, (**A**) incubation time, and (**B**) pH value.

The effect of incubation time on photocurrent was investigated. We dripped 10 μL 50 nM of T-DNA on the constructed electrode and incubated for diverse times. The results are shown in Figure 5A exhibit that the photocurrent reduced to 1.2 μA when the incubation time was up to 60 min. After the incubation time exceeded 60 min, the photocurrent remained unchanged with the incubation time increasing. This indicates that the T-DNA completely combined with P-DNA for 60 min. Therefore, the incubation time of 60 min was selected.

The pH value of PBS solution used in the experiment was 7.06, and the pH value was adjusted with dilute hydrochloric acid and sodium hydroxide solution to analyze the effects of pH values on photocurrent. After 10 μL 50 nM T-DNA was drip-coated and connected to the electrode, the PEC characterization was performed at pH values of 6.5, 6.8, 7.06, 7.2, and 7.5, respectively, as shown in Figure 5B. The analysis shows that, when the pH value was 7.06, the PEC performance was the best, and thus the PBS solution with a pH value of 7.06 was selected as the electrolyte solution.

### 3.6. The Detection of Target DNA(T-DNA)

**Figure 6:**
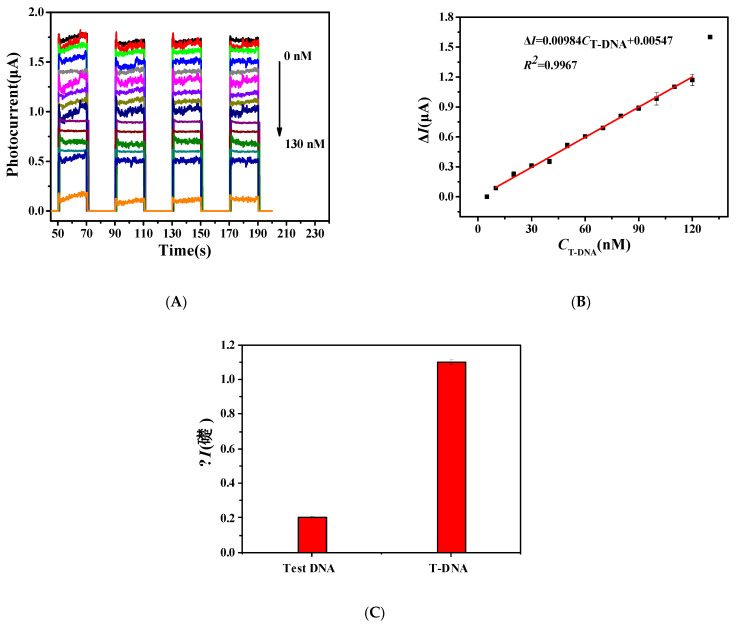
(**A**) Photocurrent and (**B**) linear fitting of the constructed electrode after adding different concentrations of T-DNA. (**C**) Photocurrent comparison after adding different DNA.

The composite electrode was coated with 10 μL T-DNA solution in different concentrations. After incubation at 37 °C for 1 h, the constructed electrodes were washed with Tris-HCl and characterized in PBS solution with pH = 7.06. As shown in Figure 6A, the photocurrent did not change significantly after the addition of 10 μL 5 nM T-DNA, but it decreased gradually after the addition of 10 μL 10–120 nM T-DNA, and the decreased interval as the same. However, after adding 10 μL 130 nM T-DNA, the photocurrent value no longer decreased uniformly but displayed a large decrease, indicating that the concentration would be beyond the range of linear detection. 

According to the data in Figure 6A, the detection limit was further calculated by linear fitting, as shown in Figure 6B. The result of linear fitting shows that the photocurrent difference had good linearity with the concentration of T-DNA in the range of 10–120 nM. The linear equation expression is ∆*I* = 0.00984C_T-DNA_ + 0.00547 (*R*^2^ = 0.996), and the detection limit is 8.39 nM. The sensitivity of the constructed PEC biosensors is relatively good and has a large detection range. Moreover, it can be seen from the figure that the photocurrent value remains unchanged after several cycles, demonstrating that the constructed PEC detection technology has good stability.

### 3.7. Method Specificity Test

In order to verify the specificity of the prepared PEC biosensors, 10 μL 100 nM Test DNA was added and treated with the same condition of T-DNA. The result is shown in Figure 6C. After adding a high concentration of Test DNA for completely non-complementary pairing, the photocurrent did not decrease significantly, while the photocurrent of the fully complementary pairing control group at the same concentration reduce to 1.1 μA, indicating that the constructed PEC detection method had good specificity for T-DNA. In the future, the ITO substrate could be replaced by a flexible material, such as conductive polymers [25], and then the prepared PEC biosensors could be used as portable detection devices.

## 4. Conclusions

In this work, we proposed a PEC detection technology based on TiO_2_. Using the hydrothermal method, we used citric acid, polyoxyethylene diamine, and polyethylene polyamine as precursors. After dialysis, CDs with uniform particle size and good fluorescence performance were obtained, and their surfaces were rich in -NH_2_ and -COOH. Using black phosphorus crystal powder as a raw material, BPQDs with a uniform particle size were prepared by the heating reflux method. 

We found that CDs and BPQDs enhanced the light absorption, and the BPQDs could improve the electron-hole separation; thereby, the nanocomposites successfully improved the PEC performance of TiO_2_. The photocurrent of the nanocomposites was up to 2.7 μA, which is about triple that of TiO_2_. Moreover, the photo-response of the nanocomposites was stable and quick. The rich surface group of the nanocomposite was conducive to binding to P-DNA, which was modified with -NH_2_. 

The results show that the incubation time and pH value can affect the PEC detection. Utilizing complementary base pairing, the specific detection of T-DNA was realized. The linear range was 10–120 nM, and the detection limit was 8.39 nM. The constructed photoelectric chemical detection technology demonstrated good sensitivity, a wide detection range, and good specificity, and the purpose of detecting different target DNA can be achieved by transforming different P-DNA. In addition, its application in detecting other substances can be further developed in the future. 

## Supplementary Materials

The following are available online at https://www.mdpi.com/article/10.3390/mi12121523/s1. Figure S1. Schematic illustration of the operation system for the PEC detection. Figure S2. TEM image of the TiO_2_@CDs@BPQDs nanocomposites.
