# Supplementary material for: Construction of Photoelectrochemical DNA Biosensors Based on TiO2@Carbon Dots@Black Phosphorous Quantum Dots
Source: Micromachines (Basel). 2021 Dec 8;12(12):1523. doi: 10.3390/mi12121523 (PMC8707531; doi:10.3390/mi12121523)
Supplement: Supplementary file 1 [file micromachines-12-01523-s001.zip › micromachines-1474920-supplementary.pdf]

micromachines

Construction of photoelectrochemical DNA biosensors based on TiO_2@carbon dots@black phosphorous quantum dots

Kai Song[1], Jianwei Lin[1,2], Yafeng Zhuang[1,2], Zhizhong Han[1,2,*] and Jinghua Chen[1,2]

[1]School of Pharmacy, Fujian Medical University, Fuzhou, Fujian 350122, China
[2]Fujian Key Laboratory of Drug Target Discovery and Structural and Functional Research, Fuzhou 350122, China
* Correspondence: Corresponding author
E-mail: zzhan@fjmu.edu.cn

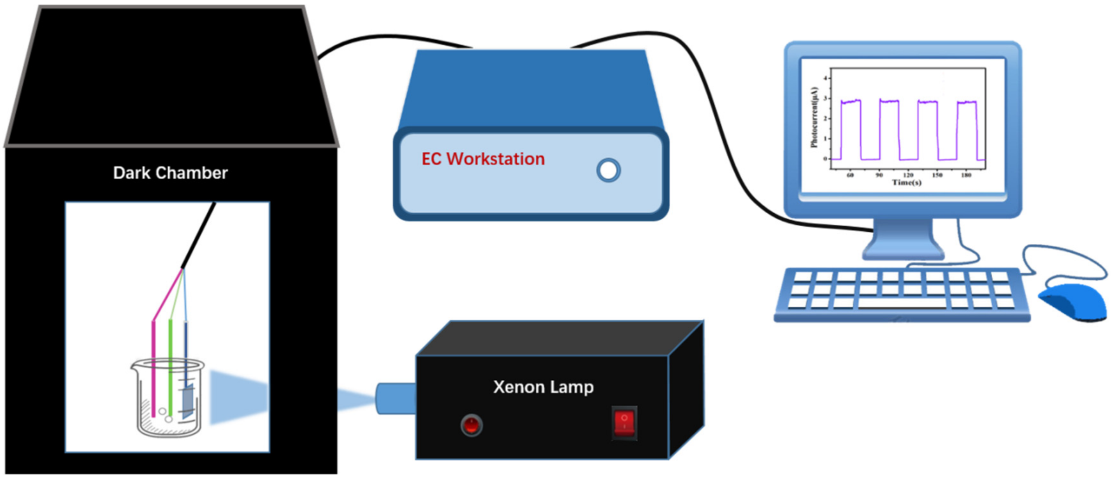

Figure S1. Schematic illustration of the operation system for the PEC detection.

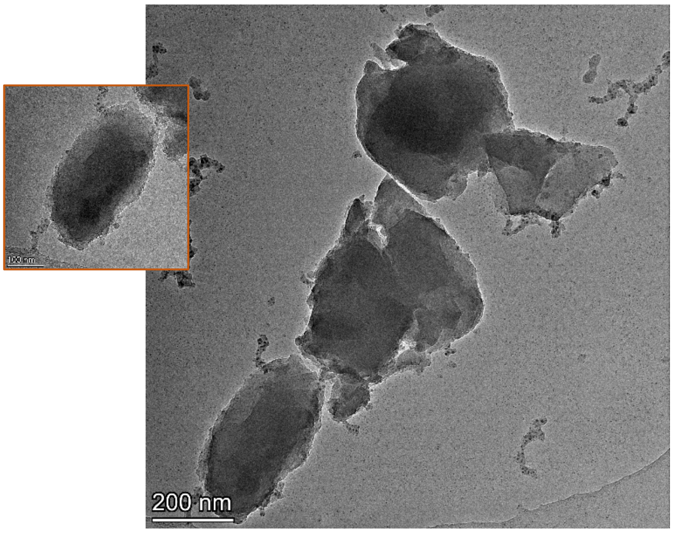

Figure S2. TEM image of the TiO_2@CDs@BPQDs nanocomposites.
